# Carbonic Anhydrases: Nature’s Way to Balance CO_2_ Concentration

**DOI:** 10.21767/2471-8084.100008

**Published:** 2015-12-16

**Authors:** Mayank Aggarwal, Robert McKenna

**Affiliations:** 1 Division of Biology and Soft Matter, Oak Ridge National Laboratory, Oak Ridge, TN 37831, USA; 2 Department of Biochemistry and Molecular Biology, College of Medicine, University of Florida, Gainesville, FL 32610, USA

The carbonic anhydrases (CAs; EC 4.2.1.1) are a family of structurally diverse (in both fold and oligomeric state), yet efficient metalloenzymes that catalyze the reversible hydration of CO_2_ and bicarbonate. They are categorized into five distinct classes (α, β, γ, δ, and ζ). Among these, the αCAs are found primarily in vertebrates, the βCAs are dominantly expressed in higher plants and some prokaryotes, while γCAs are present only in archaebacteria, and the δ and ζ classes have thus far been only isolated in diatoms. These ubiquitous enzymes equilibrate the reaction between three simple chemical molecules: CO_2_, bicarbonate, and protons; hence, they have important roles in ion transport, acid-base regulation, gas exchange, photosynthesis, and CO_2_ fixation (Figures 1A–1C) [1].

As such, structural studies of how this family of enzyme binds CO_2_ and convert it to bicarbonate may help in the understanding and designing of bio-industrial technologies for carbon sequestration. Recently, high-pressure cryo-crystallography studies have been successful in “trapping” CO_2_ in the active sites of an αCA and a βCA (Figures 1D and 1E) [2,3]. Note, Figure 1E shows a model of a γCA-CO_2_ complex which is based on the structural similarities observed between the αCA and βCA-CO_2_ complexes.

These studies are significant for several reasons: (1) they demonstrate a substrate (with a kcat/KM approaching diffusion controlled limits of 108 M-1s-1) can be captured in an enzyme active site, (2) they show the mechanistic orientation of CO_2_ in a hydrophobic pocket, positioned and poised for the nucleophilic attack of a zinc-bound hydroxide to produce bicarbonate, but most importantly (3) they demonstrate that structurally distinct enzyme folds have evolutionarily converged to create very similar active sites that maintain CO_2_ and bicarbonate concentrations in cells [4].

## Figures and Tables

**Figure 1:**
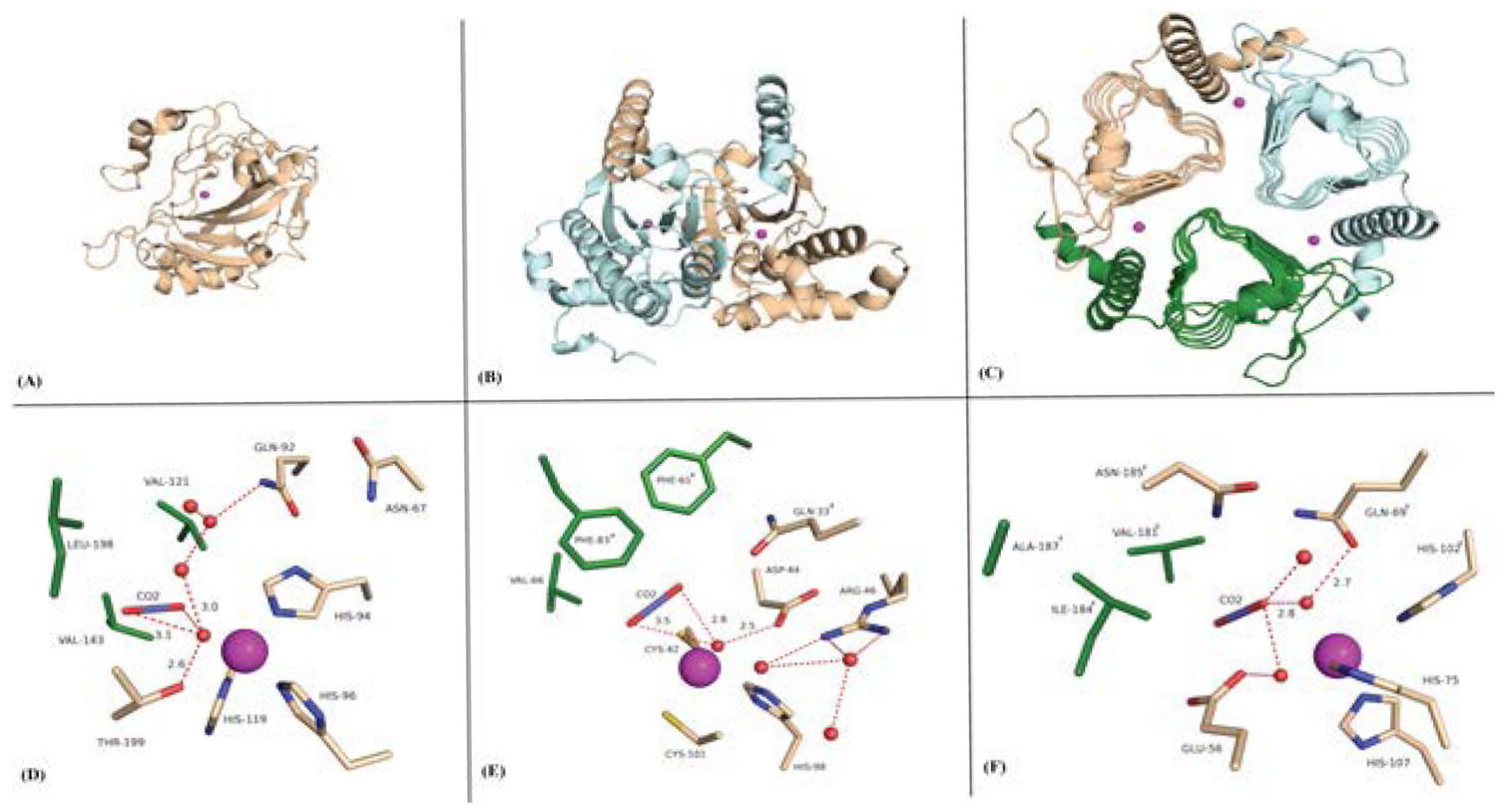
Ribbon representation of (A) monomeric αCA [PDB ID: 3D92, beige] [2], (B) dimeric βCA [PDB ID: 5BQ1, beige and blue] [3], and (C) trimeric γCA [PDB ID: 3KWC, beige, blue, and green] [4]. Stick representation of the active site-CO_2_ trapped (D) αCA, (E) βCA, and (E) γCA. Green (hydrophobic) and beige (hydrophilic) amino acids are as labeled. Active site Zn is shown as a magenta sphere.
